# Notch signaling inactivation by small molecule γ-secretase inhibitors restores the multiciliated cell population in the airway epithelium

**DOI:** 10.1152/ajplung.00382.2022

**Published:** 2023-04-11

**Authors:** Eszter K. Vladar, Koshi Kunimoto, Laura S. Rojas-Hernandez, Jacquelyn M. Spano, Zachary M. Sellers, Nam Soo Joo, Riley A. Cooney, Jeffrey D. Axelrod, Carlos E. Milla

**Affiliations:** ^1^Division of Pulmonary Sciences and Critical Care Medicine, Department of Medicine, University of Colorado School of Medicine, Aurora, Colorado, United States; ^2^Department of Pathology, Stanford University School of Medicine, Stanford, California, United States; ^3^Center for Excellence in Pulmonary Biology, Stanford University School of Medicine, Stanford, California, United States; ^4^Division of Pediatric Gastroenterology, Hepatology, and Nutrition, Department of Pediatrics, Stanford University School of Medicine, Stanford, California, United States

**Keywords:** airway epithelium, cilia, cystic fibrosis, γ-secretase inhibitor, Notch

## Abstract

Multiciliated cell loss is a hallmark of airway epithelial remodeling in chronic inflammatory airway diseases including cystic fibrosis (CF), asthma, and chronic obstructive pulmonary disease. It disrupts mucociliary clearance, which fuels disease progression. Effective clearance requires an optimal proportion of multiciliated and secretory cells. This is controlled by Notch signaling such that between two adjacent cells the one that activates Notch becomes a secretory cell and the one that avoids Notch activation becomes a multiciliated cell. Consequently, blocking Notch by a small molecule inhibitor of the γ-secretase enzyme that cleaves the Notch receptor for signal activation directs differentiation toward the multiciliated lineage. Thus, γ-secretase inhibitor (GSI) treatment may alleviate multiciliated cell loss in lung disease. Here, we demonstrate the therapeutic restoration of multiciliated cells by the GSI LY450139 (semagacestat). LY450139 increased multiciliated cell numbers in a dose-dependent manner in healthy primary human nasal epithelial cells (HNECs) during differentiation and in mature cultures, but not when applied during early epithelialization of progenitors. LY450139 did not impact stem cell proliferation. Basal and apical administration were equally effective. In healthy adult mice, LY450139 increased multiciliated cell numbers without detectible toxicity. LY450139 also increased multiciliated cells and decreased excess mucus secretory cells in CF HNECs and IL-13 remodeled healthy HNECs. LY450139 normalized multiciliated cell numbers in CF HNECs without interfering with the activity of CFTR modulator compounds. In summary, we demonstrate that GSI administration is a promising therapeutic to restore multiciliated cells and potentially improve epithelial function in a wide range of chronic lung diseases.

**NEW & NOTEWORTHY** Our findings show that low-dose, short-term topical or systemic γ-secretase inhibitor treatment may lead to restoration of multiciliated cells without toxicity and potentially improve epithelial function in a wide range of chronic lung diseases.

## Graphical abstract

**Figure:**
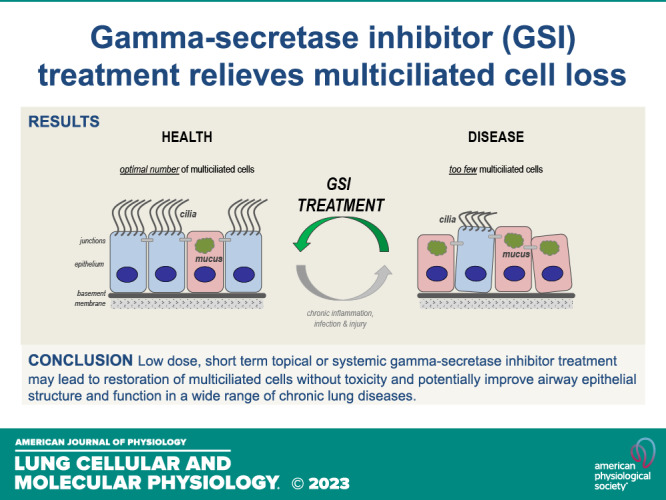

## INTRODUCTION

The airway epithelium is a patchwork of luminal multiciliated and secretory cells and an underlying layer of basal stem cells. It safeguards against inhaled pathogens, allergens, toxins, and debris by acting as a physical barrier, participating in immune surveillance and response, and via the mucociliary clearance of contaminants by the directional motility of cilia. In case of injury, the healthy epithelium can promptly and completely regenerate. In chronic inflammatory airway diseases including cystic fibrosis (CF), asthma, chronic obstructive pulmonary disease (COPD), and chronic rhinosinusitis (CRS), cycles of infection, injury, and inflammation lead to structural and functional epithelial changes termed remodeling (1–4). Remodeling results in defective mucociliary clearance and abnormal barrier, regenerative and immune functions. It is a key driver of symptoms, disease progression, and mortality. Yet, treatments do not directly target or reverse it.

Damaged and dysfunctional cilia and multiciliated cell loss are readily detectible both histologically and functionally in remodeled airways (1, 5). Cilia and multiciliated cells are directly injured by pathogens, neutrophil elastase, and cigarette smoke components. More importantly, proinflammatory cytokines abundant in diseased airways such as IL-13 and IL-1β are potent drivers of mucous cell hyperplasia at the expense of multiciliated cells (6, 7). Diseased airways also contain extensive regions of poorly differentiated epithelium, possibly due to a maladaptive regenerative response to frequent injury (8). The extent of multiciliated cell loss and mucociliary dysfunction correlates with disease severity in CF, asthma, COPD, and CRS (9–12). In CF, it remains detectable despite treatment with highly effective modulator therapy (HEMT) that corrects the underlying dysfunction in the cystic fibrosis transmembrane conductance regulator (CFTR) ion channel (13). Thus, restoration of the optimal proportion of functional multiciliated cells remains an unmet therapeutic goal.

The Notch pathway is critical to maintain the proper proportion of multiciliated and secretory cells (14, 15). Notch signaling involves the binding of a membrane-tethered JAGGED or DELTA family ligand on one cell to a NOTCH family receptor on the adjacent cell (16). NOTCH undergoes extensive enzymatic cleavage. A final, ligand-dependent intramembrane cleavage ultimately releases its intracellular domain to act as a transcriptional regulator of target genes in the nucleus. This final cleavage by the γ-secretase enzyme is therefore necessary for Notch signaling, and as such, small molecule γ-secretase inhibitors (GSIs) are potent Notch pathway inhibitors (17). Notch controls airway epithelial differentiation such that between two adjacent cells the one that activates the Notch pathway becomes a secretory cell and the cell that avoids Notch activation becomes a multiciliated cell (14, 15). There is also evidence that basal stem cells can engage in Notch signaling (18). The Notch pathway is frequently aberrantly regulated in chronic lung disease (19). NOTCH3 is upregulated in COPD and asthma, and it contributes to remodeling by driving mucous cell hyperplasia (20, 21). Notch ligands have also been implicated in generating a Type 1 versus Type 2 inflammatory response in allergic asthma, although the mechanism is not clear (22).

Events linking Notch ligand presentation and downstream motile ciliogenesis in nascent multiciliated cells are only partially understood. However, Notch inhibition increases, while Notch activation blocks, multiciliated cell formation both in in vivo and primary culture models (14, 15, 23, 24). Consequently, manipulation of multiciliated cell numbers by Notch pathway inhibition is a promising therapeutic option to alleviate multiciliated cell loss in remodeled airways. We previously showed that upon treatment with DAPT, a GSI tool compound commonly used in research, multiciliated cell numbers in CF and CRS derived primary human nasal epithelial cultures are restored to levels comparable to those of healthy donor cultures (24). Importantly, restoring multiciliated cells also improved barrier function and regeneration after an epithelial scratch wound.

Multiple GSIs have been developed to therapeutically block Notch signaling to treat a variety of cancers, graft versus host disease, endometriosis, and to restore auditory hair cells (17, 25, 26). Separately, GSIs have been pursued in Alzheimer’s disease based on the premise that γ-secretase-dependent accumulation of amyloid precursor protein cleavage products in the brain is pathogenic (27). As of now, at least seven GSIs have been tested in clinical trials (26). Early trials faced challenges due to on-target (Notch inhibition-dependent) gastrointestinal toxicity in the setting of long-term (weeks to months), high-dose, systemic GSI treatment deemed necessary to penetrate the blood-brain barrier or to combat solid tumors. Therapeutic Notch inhibition is still actively pursued for indications other than Alzheimer’s disease. GSIs remain an attractive therapeutic modality especially if localized administration, reduced dosing, or a shorter treatment course can be achieved.

Here, we demonstrate that LY450139 (semagacestat) (28), a GSI that has been studied extensively in preclinical models and clinical trials efficiently mitigates multiciliated cell loss. Basal and apical administration of LY450139 increased multiciliated cell numbers in a dose-dependent manner in differentiating and homeostatic cultures of healthy primary human nasal epithelial cells (HNECs). LY450139 had no detrimental effect on stem cell proliferation or function. In healthy adult mice, low-dose, short-term LY450139 treatment increased multiciliated cell numbers without detectible toxicity. LY450139 also increased multiciliated cells and decreased mucous cell hyperplasia in HNECs remodeled by IL-13. Finally, we demonstrate that LY450139 normalized multiciliated cell numbers in CF HNECs without interfering with HEMT activity. Our data show that GSI administration is a promising therapeutic to restore multiciliated cells and potentially improve epithelial function in a wide range of chronic lung diseases.

## MATERIALS AND METHODS

### Primary Human Nasal Epithelial Cell Cultures

Primary human nasal epithelial cells were obtained by brush biopsy of the inferior turbinates with written informed consent from subjects recruited via the Cystic Fibrosis Center at Stanford University (Human Subjects Protocol No. 42710). Primary air-liquid interface (ALI) culture of nasal epithelial cells from *passage 0* (P0) or P1 basal stem cells was carried out as we previously described (24, 29). Briefly, cells are removed from cytobrushes with gentle enzymatic digestion. P0 HNEC cultures are initiated by seeding freshly isolated cells onto Collagen I-coated Transwell filters. P1 HNEC cultures are initiated from basal cells that were first expanded on Collagen I-coated plastic dishes in proliferation medium supplemented with Y-27632 (ROCK inhibitor, 10 µM), DMH-1 (BMP inhibitor, 1 µM), A-83-01 (TGF-β inhibitor, 1 µM), and CHIR-99021 (Wnt agonist, 1 µM) (30), all from Selleckchem. HNECs seeded onto Transwells (Corning, 3470) are initially cultured submerged in proliferation medium until confluency, then lifted to ALI (considered as ALI + 0 days of culture) by supplying differentiation medium only from the bottom compartment. HNECs are considered mature at ALI + 21 days of culture. Healthy and CF HNECs used in the study were cultured in homemade media (24). CF HNECs used for Ussing chamber measurements only were cultured in Pneumacult Ex+ (proliferation) and Pneumacult ALI (differentiation) media (Stemcell). GSI compounds (DAPT, LY450139, PF-03084014, RO-4929097, and MK-0752) were obtained from Selleckchem and reconstituted in DMSO then diluted in culture medium (HNEC treatment) or saline (mouse IP injection). Healthy HNECs were treated with 10 ng/mL recombinant human IL-13 (RnD Systems). GSI-treated and control HNEC lysates were pooled from three replicate cultures for Western blot analysis. For small molecules, see Supplemental Table S1 (all Supplemental material is available at https://doi.org/10.6084/m9.figshare.22304557).

### Wholemount Immunofluorescence and Scanning Electron Microscopy of HNEC Cultures

For wholemount immunofluorescence, HNECs were fixed in −20°C methanol or 4% paraformaldehyde for 10 min as previously described (31). Transwell filters were cut out of the plastic supports and placed in a humid chamber for staining. Samples were blocked in 10% normal horse serum and 0.1% Triton X-100 in PBS and incubated with primary antibodies for 1–2 h, then with Alexa dye conjugated secondary antibodies (Thermo Fisher) for 30 min at room temperature. Filters were mounted in Mowiol mounting medium containing 2% N-propyl gallate (Sigma). Samples were imaged with a Leica SP8 or Zeiss LSM 900 confocal microscope. For antibodies and fixation conditions, see Supplemental Table S1. For SEM, Transwell filters were fixed in 2% glutaraldehyde, 4% paraformaldehyde in 0.1 M NaCacodylate buffer, pH 7.4 at 4°C overnight. Samples were osmicated, dehydrated, dried with a Tousimis Autosamdri-815 critical point dryer. Samples were mounted luminal side up, sputter coated with 100 Å layer of Au/Pd. Images were acquired with a Hitachi S-3400N VP-SEM microscope operated at 10–15 kV, with a working distance of 7–10 mm and using secondary electron detection.

### Ciliary Length and Ciliary Beat Frequency (CBF) Measurement in HNEC Cultures

ALI + 21 days HNECs were washed with warm PBS to remove mucus, then the Transwell filter was gently cut out of the plastic support. The filter was carefully folded along the center such that the folded edge exposed the mucosal surface for the evaluation of beating cilia. The folded filter was placed on a microscope slide inside a 100 µm mask, 100 µL of warm medium was added to maintain humidity, and a coverslip was immediately placed over the filter to seal the preparation. The slide was then placed on a heated stage at 37°C and imaged with a digital video microscope fitted with a Keyence VW-9000 series high-speed camera (Keyence). Images were captured at ×2,000 and 500 fps on multiple areas per filter. Average CBF (Hz) was calculated from kymographs generated from the high-speed videos. Ciliary length was measured from still images of the recording where the cilia were orthogonal to the apical surface.

### Mouse Husbandry and GSI Treatment in Mice

*Foxj1/EGFP* mice have been previously described (32, 33). 10–40 wk age-matched male and female *Foxj1/EGFP* mice were treated with LY450139, DAPT, or vehicle control by intraperitoneal (IP) administration. In the first experiment, 0.1 mg/kg, or 1 mg/kg LY450139, 10 mg/kg DAPT or saline vehicle was administered twice daily for three consecutive days, then the mice were euthanized on *day 7*. In the second experiment, 1 mg/kg LY450139 or saline vehicle was administered once daily for five consecutive days per week for 3 wk, then the mice were euthanized on *day 30*. Body weight was monitored as a surrogate measure of gastrointestinal toxicity. See Supplemental Fig. S3*A* for more information. For both studies, the lungs were cryoembedded en bloc in OCT compound (Thermo Fisher). Cryosections were formalin-fixed, then labeled with anti-GFP and acetylated α-Tubulin antibodies and stained with DAPI to mark nuclei. Images were acquired using a Leica SP8 confocal microscope and analyzed by three independent blinded investigators as follows. In airways of approximately uniform size viewed in cross section, cells were first identified by DAPI signal, which provided the total luminal cells per airway metric. Cells were then scored either as multiciliated cells (GFP positive) or nonciliated cells (GFP negative). Anti-acetylated α-Tubulin signal was used as an additional identifying feature for multiciliated cells. Data are presented as multiciliated cells per total luminal cells. See Supplemental Fig. S3*B* for example image. All procedures involving animals were approved by the Institutional Animal Care and Use Committee of Stanford University School of Medicine in accordance with established guidelines for animal care.

### CFTR Activity Measurement in CF HNEC Cultures

CF HNECs were differentiated from ALI + 0 to 21 days in the presence or absence of 125 nM LY450139 with and without 3 µM elexacaftor and 3 µM tezacaftor (Selleckchem). Transwells were mounted in an Ussing chamber for electrophysiological short circuit current (I_sc_) measurements using standard methods (34). Solutions in the serosal and mucosal baths were prepared so that a chloride gradient was established between both sides. After stable baseline current recordings were obtained, agonists were added in the following order to the apical side: amiloride (10 µM) to block sodium channel activity, IBMX and forskolin (10 µM) to stimulate CFTR through increased cAMP, ivacaftor (10 µM) to potentiate CFTR activity, and CFTR_inh_-172 (20 µM) to block CFTR current. For each agonist, signals were monitored until a plateau in current was noted before adding the next agonist. The delta-I_sc_ in response to CFTR_inh_-172 was used as the chief readout for CFTR-mediated chloride transport.

## RESULTS

### Selection of a Small Molecule γ-Secretase Inhibitor to Increase the Proportion of Multiciliated Cells in Healthy Airway Epithelia by Inhibition of Notch Signaling

**Figure 1.**
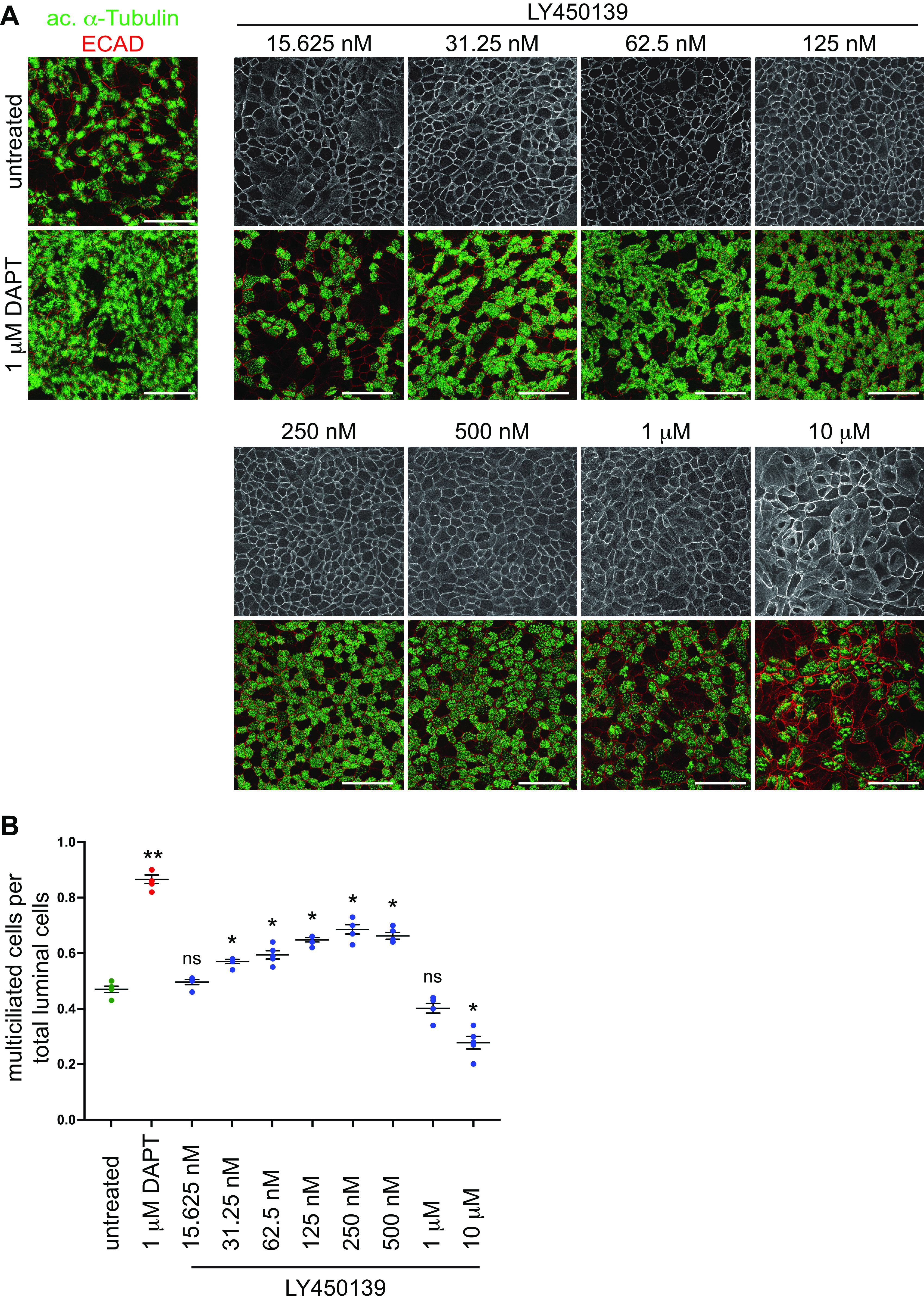
GSI treatment induces multiciliated cell formation. *A*: primary human nasal epithelial cells were treated during differentiation (ALI + 0 to +21 days) with a range of concentrations of LY450139 and labeled at ALI + 21 days with anti-acetylated α-Tubulin (green) and ECAD (red) antibodies to mark cilia and epithelial junctions. High concentration (10 µM) of LY450139 disrupted the epithelial junctions, but lower concentrations lead to a dose-dependent increase in multiciliated cell numbers similar to DAPT. Scale bar, 50 µm. *B*: quantitation of data shown in *A*. One-way ANOVA, ns, not significant, *P < 0.01, **P < 0.001. ALI, air-liquid interface; GSI, γ-secretase inhibitor.

The γ-secretase inhibitor (GSI) DAPT, is known to block Notch signaling and increase the proportion of multiciliated cells in an in vitro reconstituted respiratory epithelium (23, 24). Although this effect may be beneficial as a therapeutic, DAPT is not suitable for human use. We therefore sought to identify GSIs for potential therapeutic application among small molecules previously used in clinical trials that were developed to have acceptable drug-like properties. Several candidate GSIs were selected based on literature searches (17, 26) and their commercial availability from a trusted manufacturer: LY450139 (semagacestat), PF-03084014 (nirogacestat), RO-4929097 and MK-0752. Healthy human nasal epithelial cell primary cultures (HNECs) were grown at air-liquid interface (ALI) in homemade culture medium to support the consistent differentiation of multiciliated cells as ∼45–55% of the luminal cells, similar to the in vivo airways (24) (Fig. 1*A*, Supplemental Fig. S1*A*). The use of homemade culture medium is important, as cultures grown in commercially available Pneumacult medium often contain 85%–95% multiciliated cells at the luminal surface (Supplemental Fig. S4*A*). HNECs were treated with a low and high dose (based on IC_50_ for Notch inhibition, Selleckchem) of each compound during the entire 21 days of ALI differentiation (ALI + 0 to 21 days). The fraction of mature multiciliated cells was quantitated using wholemount anti-acetylated α-Tubulin antibody (marks ciliary axonemes) labeling. We found that, like DAPT, all GSIs we tested were able to induce the formation of extra multiciliated cells (Supplemental Fig. S1*A*; https://doi.org/10.6084/m9.figshare.22304557). DMSO control treatment had no effect on multiciliated cell number (Supplemental Fig. S1*A*).

Here, we further characterize LY450139, as of the four candidates, it is the most extensively studied GSI in both in vitro and in vivo models as well as clinical trials (28). We determined that LY450139 treatment was able to effectively increase the proportion of multiciliated cells at as low as 31.25 nM concentration and begins to plateau at around 125 nM (Fig. 1, *A* and *B*). At doses in the µM range, all GSIs, including DAPT disrupted epithelial structure and multiciliated cell formation (Fig. 1, Supplemental Fig. S1). We show that GSI treatment at doses in the nM range led to the addition of structurally and functionally normal multiciliated cells. It had no effect on ciliary length (Supplemental Fig. S1*C*), overall morphology (Supplemental Fig. S2), or the number of cilia per cell (not shown). GSI treatment slightly increased ciliary beat frequency, although it remained within the range reported for human sinonasal cilia (35) (Supplemental Fig. S1*C*). As expected, both DAPT and LY450139 treatment led to a drastic reduction in cleaved NOTCH1 confirming that they act as inhibitors of Notch signaling in HNECs (Supplemental Fig. S1*D*).

### Establishing the Effective GSI Treatment Time Window and Mode of Exposure in Healthy Airway Epithelia

**Figure 2.**
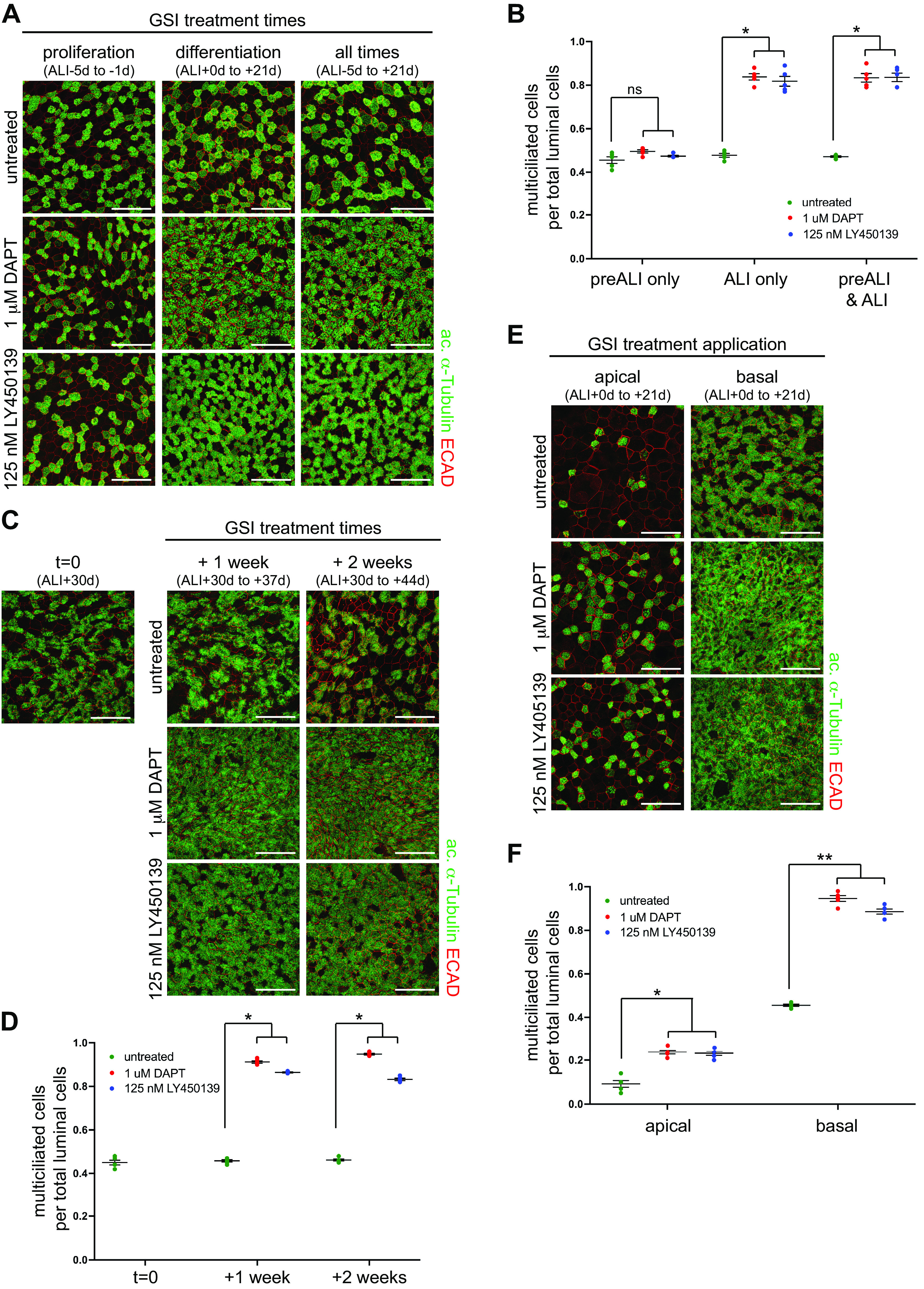
GSI treatment induces multiciliated cell formation in differentiating and mature airway epithelia. *A*: primary human nasal epithelial cells were treated with DAPT and LY450139 during pre-ALI only (ALI-5 to -1 day = proliferation), during ALI only (ALI + 0 to +21 days = differentiation) or continuously during pre-ALI and ALI (ALI-5 days to +21 days = all times) and labeled at ALI + 21 days with anti-acetylated α-Tubulin (green) and ECAD (red) antibodies. Only GSI treatment during differentiation and all times increased multiciliated cell numbers. GSI treatment during pre-ALI (proliferation only and all times treatments) had no detrimental effect on multiciliated cells or overall epithelial structure. Scale bar, 50 µm. *B*: quantitation of data shown in *A*. One-way ANOVA, ns, not significant; *P < 0.0001. *C*: mature (ALI + 30 days) primary human nasal epithelial cells were treated with DAPT and LY450139 for one (ALI + 30 to +37 days) or 2 wk (ALI + 30 to +44 days), then labeled with anti-acetylated α-Tubulin (green) and ECAD (red) antibodies. GSI treatment induces the formation of additional multiciliated cells in mature cultures, while untreated cultures do not form any more multiciliated cells. Scale bar, 50 µm. *D*: quantitation of data shown in *C*. One-way ANOVA, *P < 0.0001. *E*: primary human nasal epithelial cells were treated with DAPT and LY450139 during differentiation only (ALI + 0 to +21 days) from either the apical or basal surface, then labeled at ALI + 21 days with anti-acetylated α-Tubulin (green) and ECAD (red) antibodies. GSI treatment induces multiciliated cell formation via both apical and basal application. Apical treatment abrogates the air-liquid interface, which results in the poor epithelial structure and multiciliated cell differentiation in the untreated cultures, which is partially rescued by GSI treatment. Scale bar, 50 µm. *F*: quantitation of data shown in *E*. One-way ANOVA, *P < 0.01, **P <0.001. ALI, air-liquid interface; GSI, γ-secretase inhibitor.

HNEC primary culture comprises an initial proliferative phase when basal stem cells establish the epithelial layer under submerged culture conditions (pre-ALI), followed by differentiation induced, in part, by lifting to ALI (29). Thus, HNECs can be used to test GSI response in both regenerative and homeostatic settings. Notch signaling between luminal cells controls epithelial cell fate, but it is also active in basal cells and regulates their differentiation and survival (14, 18, 36). Thus, we asked if the GSIs LY450139 or DAPT added during the proliferative pre-ALI phase only or during the entire culture duration (proliferative + differentiation; pre-ALI + ALI) can induce multiciliated cell formation and if GSI treatment during stem cell proliferation has detrimental effects. Pre-ALI, we added GSIs to both the apical and basal media, and during ALI, cultures were only treated basally. We found that pre-ALI-only treatment was not sufficient to induce extra multiciliated cell formation, but similar to ALI-only, continuous (pre-ALI + ALI) treatment led to increased multiciliated cells with no apparent disruption of epithelial composition or structure (Fig. 2, *A* and *B*). This indicates that the permissive window for GSI activity is after ALI + 0 days and that continuous treatment does not adversely affect the induction of multiciliated cell formation.

To test if active differentiation is required or if treatment of already differentiated epithelium is sufficient to gain more multiciliated cells, we carried out GSI treatment in mature HNECs. To ensure that the cultures are fully differentiated, we grew cultures to ALI + 30 days. We then treated cells with LY450139 or DAPT for 1 or 2 wk. We found that both GSIs and treatment times led to the robust induction of multiciliated cell formation (Fig. 2, *C* and *D*). This indicates that GSI blockage of Notch signaling can be used to increase the fraction of multiciliated cells in both regenerating and intact epithelia.

Whereas ALI-cultured HNECs are exposed to drugs from the culture medium on their basal sides, we asked if GSI treatment applied to the apical surface is sufficient to induce multiciliated cell formation. This required the long-term culture of HNECs undergoing ALI differentiation under submerged conditions, which has been shown to suppress differentiation (7, 24). Indeed, we observed fewer multiciliated cells in submerged untreated cultures (submerged cultures were given 250 µL of apical medium, Fig. 2, *E* and *F*). However, similar to ALI cultured cells that received LY450139 or DAPT basally, apical-only LY450139 or DAPT treatment of submerged cultures was still able to approximately double the number of multiciliated cells (Fig. 2, *E* and *F*). This suggests that LY450139 could be used to increase the proportion of multiciliated cells either when administered systemically or topically to the airway epithelial surface.

### GSI Treatment Induces Multiciliated Cell Formation in the In Vivo Healthy Adult Mouse Airway Epithelium

**Figure 3.**
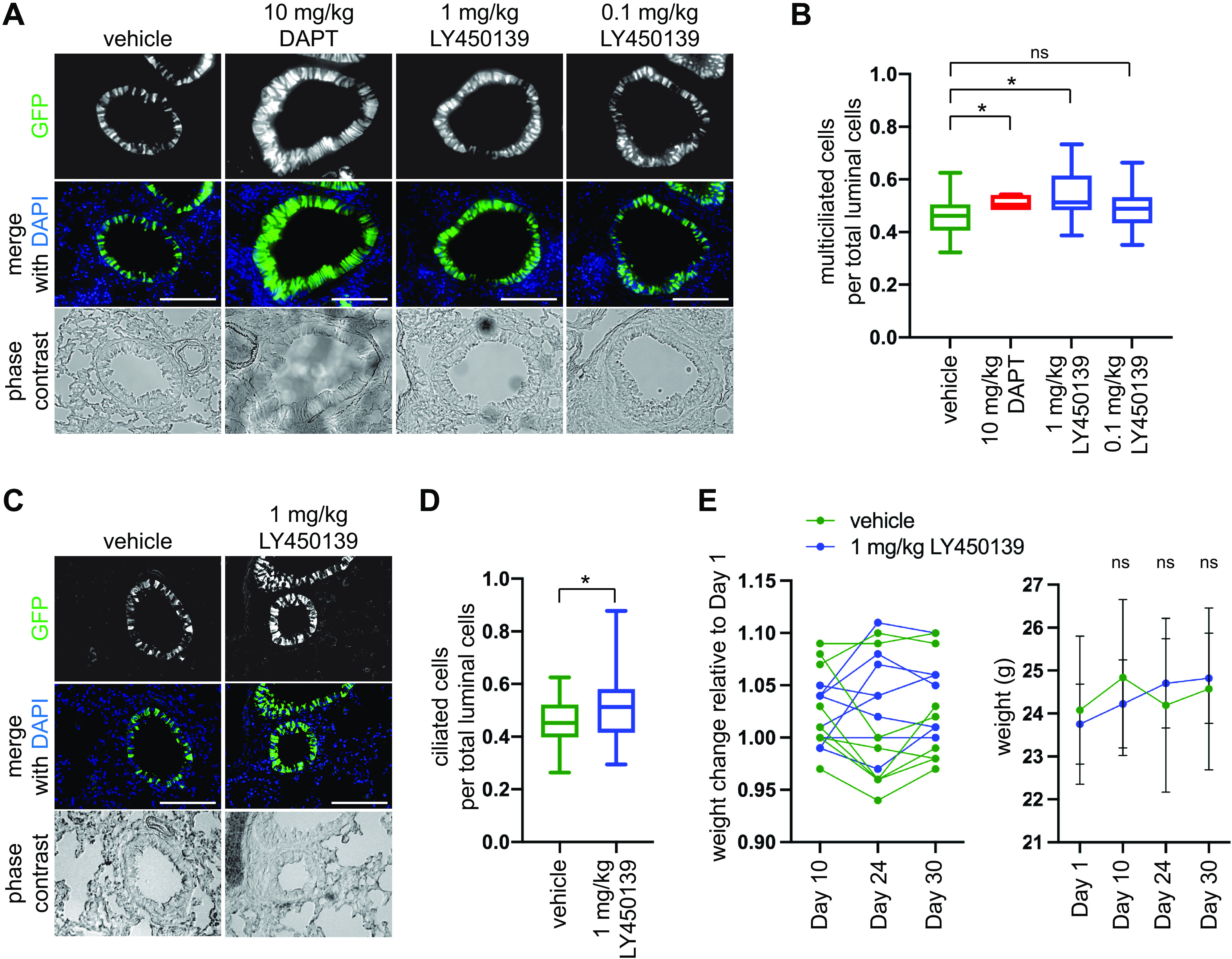
GSI treatment induces multiciliated cell formation in vivo. *A*: adult *Foxj1-EGFP* mice were treated with 10 mg/kg DAPT, 1 mg/kg or 0.1 mg/kg LY450139 or vehicle control twice daily for 3 days, then on *day 7* multiciliated cell number was quantitated in airway cross sections based on GFP fluorescence, which shows that 1 mg/kg LY450139 increased multiciliated cell number. Treatment with 0.1 mg/kg LY450139 showed a similar trend but did not reach significance (*P* = 0.09). *n* = 4 mice were treated per category. Scale bar, 100 µm. *B*: quantitation of data shown in *A*. One-way ANOVA, ns, not significant, *P < 0.05. *C*: adult *Foxj1-EGFP* mice were treated with 1 mg/kg LY450139 or vehicle control once daily for five consecutive days for 3 wk, then on *day 30* multiciliated cell number was quantitated, which shows that 1 mg/kg LY450139 increased multiciliated cell number. *n* = 8 mice were treated per category. Scale bar, 100 µm. *D*: quantitation of data shown in *C*. *T* test, *P < 0.01. *E*: mouse body weights over the course of the experiment in *C*–*D* depicted as a proportion of the *day 1* weight for individual mice (*left*) and as the average of the absolute weights (*right*), *T* test, ns, not significant. ALI, air-liquid interface; GSI, γ-secretase inhibitor.

The impact of LY450139 in mouse models of neurodegeneration has been studied extensively (27, 28). In these models, LY450139 was administered systemically for weeks to months at a time at up to 100 mg/kg. We sought to determine if a substantially lower dose administered for a shorter duration would be sufficient to induce an increase in the proportion of multiciliated cells in the intact airway epithelium of healthy adult mice. Mice were first treated with 0.1 mg/kg or 1 mg/kg of LY450139, 10 mg/kg DAPT or vehicle control twice daily for three consecutive days via intraperitoneal injection and then assessed on *day 7* (Supplemental Fig. S3*A*). To quantitate multiciliated cells, treatments were carried out in *Foxj1/EGFP* transgenic mice that express cytoplasmic GFP under the multiciliated cell-specific *Foxj1* promoter (32). Multiciliated cell number was quantitated in airways of similar size in lung tissue sections. Cells were identified by DAPI nuclear staining and were counted as multiciliated if the DAPI staining overlapped with GFP signal (Supplemental Fig. S3*B*). We found that 1 mg/kg LY450139 or 10 mg/kg DAPT lead to a modest, but statistically significant increase in the number of multiciliated cells, whereas 0.1 mg/kg LY450139 showed a trend toward more multiciliated cells that did not reach statistical significance (Fig. 3, *A* and *B*). Treatment was repeated with 1 mg/kg LY450139 or vehicle control using once daily injection for five consecutive days per week for 3 wk, which also induced an increase in the proportion of multiciliated cells (Supplemental Fig. S3*A*, Fig. 3, *C* and *D*). Importantly, GSI-treated mice did not exhibit weight loss (Fig. 3*E*), nor did we detect any other adverse event, indicating that our treatment regimen was well-tolerated.

### GSI Treatment Restores Multiciliated Cell Abundance in Airway Epithelia Depleted of Multiciliated Cells Due to Chronic Inflammatory Remodeling

**Figure 4.**
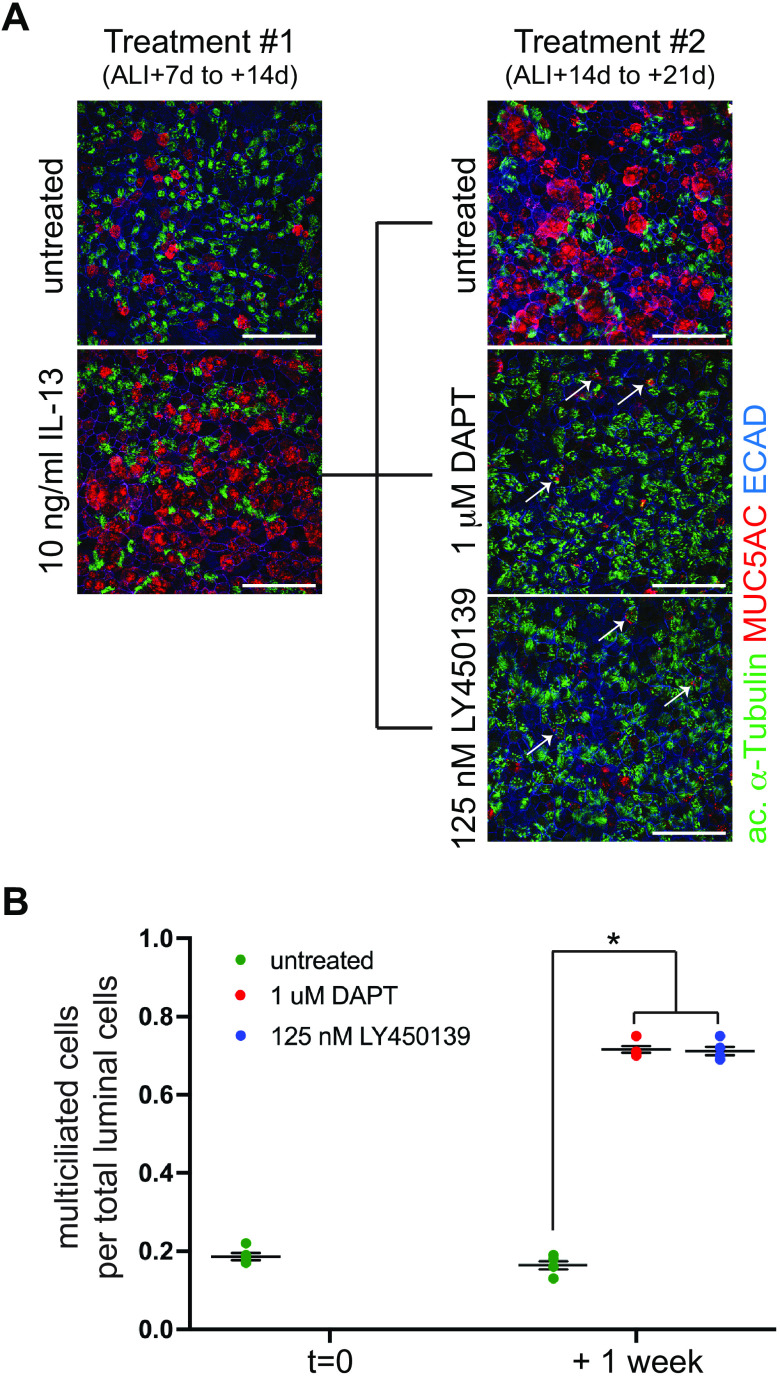
GSI treatment induces multiciliated cell formation in epithelia with mucous cell hyperplasia. *A*: primary human nasal epithelial cells were treated with recombinant human IL-13 during early epithelial differentiation (ALI + 7 to +14 days), which induced mucous cell formation at the expense of multiciliated cell differentiation as assessed by anti-acetylated α-Tubulin (green), MUC5AC (red) and ECAD (blue) antibodies. These cultures were then treated with DAPT or LY450139 for an additional week (ALI + 14 to +21 days), then assessed at ALI + 21 days. Although the untreated cells maintained the remodeled phenotype, GSI treatment induced multiciliated cell formation to levels comparable to healthy epithelia. Arrows point to acetylated α-Tubulin and MUC5AC double positive cells in GSI treated cultures. Scale bar, 50 µm. *B*: quantitation of data shown in *A*. One-way ANOVA, *P < 0.0001. ALI, air-liquid interface; GSI, γ-secretase inhibitor.

Thus far we have demonstrated that blocking Notch signaling by GSI treatment can induce multiciliated cell formation in healthy in vitro and in vivo airway epithelia. To test whether GSI treatment has the ability to restore multiciliated cells in remodeled epithelia with fewer than normal multiciliated cells, we treated healthy donor HNECs with the proinflammatory cytokine IL-13 to model mucous cell hyperplasia (7, 24). IL-13 treatment from ALI + 7 to 14 days of culture led to the formation of excess mucus secretory cells, identified by MUC5AC labeling, at the expense of multiciliated cells (Fig. 4*A*). At ALI + 14 days, the cytokine was withdrawn, and cultures were treated for 1 wk with LY450139, DAPT or DMSO control. Control cultures continued to exhibit mucous cell hyperplasia with a reduced fraction of multiciliated cells (Fig. 4, *A* and *B*). However, GSI-treated HNECs increased their proportion of multiciliated cell numbers. This was accompanied by a reduction in the proportion of mucous cells, suggesting that GSI treatment not only induces multiciliated cell differentiation, but also relieves mucous cell hyperplasia. The presence of MUC5AC and anti-acetylated α-Tubulin double positive cells, indicating an intermediate phenotype, suggests that this occurred, at least in part, through transdifferentiation of mucous to multiciliated cells upon inhibition of Notch signaling (Fig. 4*A*).

### GSI Treatment Restores Multiciliated Cell Abundance in Cystic Fibrosis Airway Epithelia and Does Not Interfere with CFTR Correction by Highly Effective Modulator Therapy

We previously showed that CF HNECs cultured from unpassaged or early passage sinonasal basal cells in homemade medium model the reduced multiciliated cell numbers focally exhibited by donor tissue (24) (Supplemental Fig. S4*A*). In homemade medium, we also showed that DAPT treatment during differentiation increases the multiciliated cell proportion in CF cultures to that of healthy HNECs (24). Here, we show that LY450139 leads to a similarly normalized proportion of multiciliated cells in CF HNECs (Supplemental Fig. S4*A*). As observed in healthy HNECs, GSI-induced multiciliated cells in CF HNECs are structurally normal, and GSI treatment in both differentiating and mature CF cultures can mitigate multiciliated cell loss (Supplemental Figs. S2, S4, *A* and *B*).

**Figure 5.**
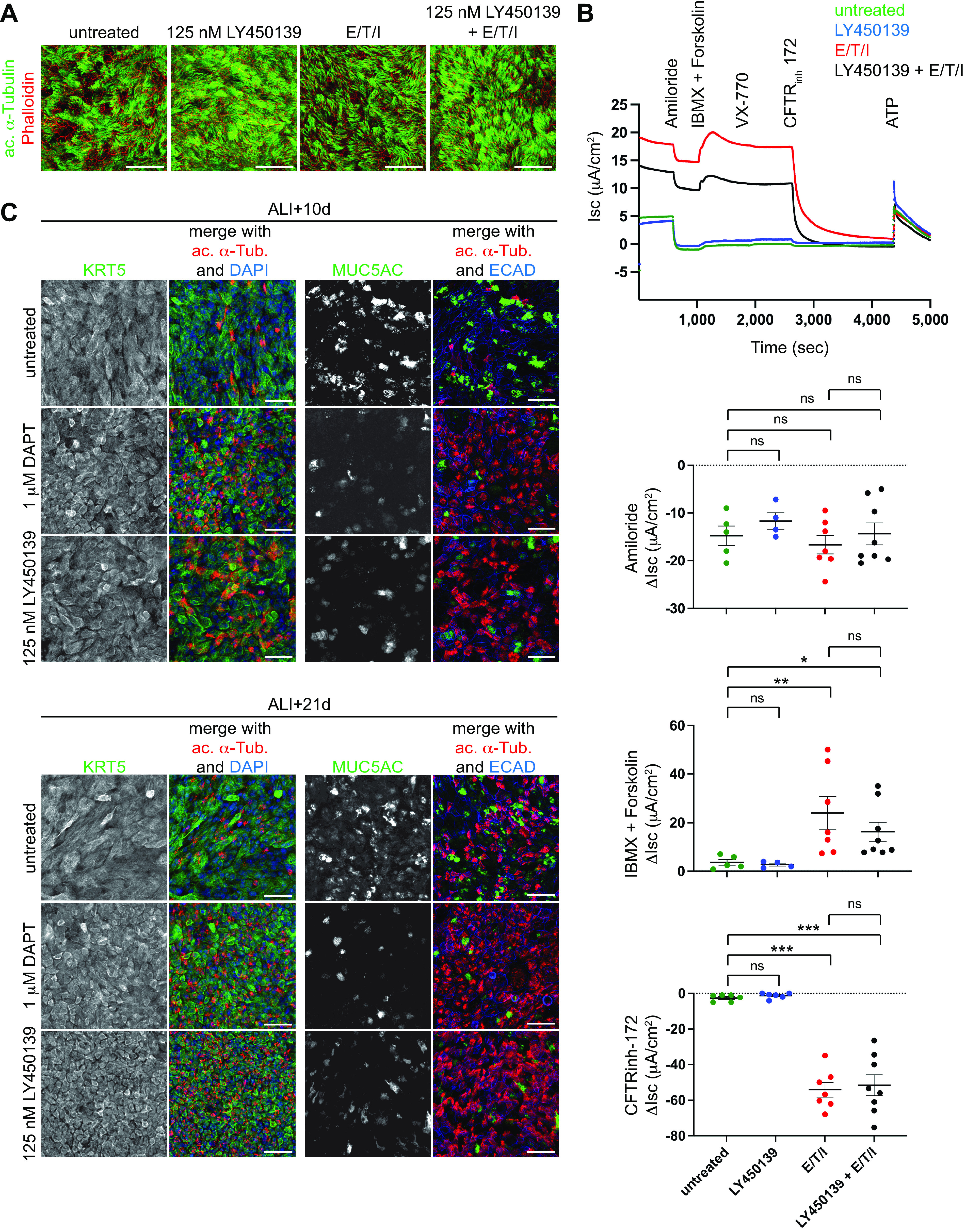
GSI treatment induces multiciliated cell formation in cystic fibrosis epithelia. *A*: primary CF nasal epithelial cells were treated with LY450139 with and without the CFTR modulators elexacaftor (VX-445) and tezacaftor (VX-661) during differentiation only (ALI + 0 to +21 days) and labeled at ALI +21 days with anti-acetylated α-Tubulin (green) and ECAD (red) antibodies. Scale bar, 50 µm. *B*: the same cells from *A* were subjected to Ussing chamber short circuit current (I_sc_) measurements to measure CFTR activity following standardized stimulation protocols. Representative Ussing chamber tracings provided for one donor (*top*), ΔI_sc_ after indicated manipulations provided for all donors (*bottom* graphs). Two-way ANOVA, *P < 0.05, **P < 0.01, ***P < 0.0001. *C*: primary CF nasal epithelial cells treated with DAPT or LY450139 during differentiation only (ALI + 0 to +21 days) and immunolabeled at ALI + 10 days (*top*) and ALI + 21 days (*bottom*) with KRT5 (green) and acetylated α-Tubulin (red) antibodies show that cultures maintain basal cells during GSI treatment (*left*) and with MUC5AC (green) and acetylated α-Tubulin (red) antibodies show that cultures decrease mucus secretory cells during GSI treatment. Scale bar, 50 µm. ALI, air-liquid interface; CF, cystic fibrosis; GSI, γ-secretase inhibitor.

Approximately 90% of people with CF (pwCF) in the United States carry at least one mutant CFTR allele that is compatible with highly effective modulator therapy (HEMT), a triple combination of the small molecules VX-445, VX-661, and VX-770 (elexacaftor/tezacaftor/ivacaftor or E/T/I) (37). The majority of pwCF are receiving E/T/I, which has led to substantial clinical improvements. Thus, it is critical that administration of a GSI as a potential adjunct therapy does not interfere with the efficacy of E/T/I. To measure a potential impact on correction of CFTR function by E/T/I, CF HNECs from four donors (Supplemental Table S2) were differentiated with and without LY450139 and E/T/I (Fig. 5*A*). Ussing chamber analysis revealed effective correction of CFTR function by E/T/I with or without LY450139 (Fig. 5*B*). We conclude that the presence of LY450139 does not interfere with E/T/I activity. We note that because the Ussing chamber assay has been optimized using the commercially available Pneumacult medium (Fig. 4*A*), the effect of LY450139 treatment on cellular composition is masked (Fig. 5*A*). In CF HNECs differentiated in homemade medium we demonstrate that both DAPT and LY450139 led to decreased mucus secretory cell numbers, but no apparent change in the basal stem cell population (Fig. 5*C*). Furthermore, we show that in contrast to published reports (38), GSI treatment in HNECs did not reduce the number of FOXI1 positive ionocytes (Supplemental Fig. S4*C*), which are thought to serve as hotspots for CFTR activity in the airway epithelium (38). Thus, a combination of GSI treatment and E/T/I has the potential to restore both normal epithelial cell composition and function in the CF airway epithelium.

## DISCUSSION

This study serves as proof of principle for the therapeutic targeting of multiciliated cell loss in chronic airway diseases by pharmaceutical grade γ-secretase inhibitors (GSIs) that block the Notch signaling pathway. We previously demonstrated that DAPT, a GSI tool compound increases multiciliated cell numbers in healthy, CF and CRS primary airway epithelial cell cultures (24). Here we show that low dose LY450139, a GSI that was developed to have improved drug-like properties and has been studied extensively in human clinical trials, also increases multiciliated cells numbers in healthy in vitro and in vivo airway epithelia and restores multiciliated cell numbers in CF cultures. We show that LY450139 increases the proportion of multiciliated cells in a dose-dependent manner, and multiciliated cells generated upon LY450139 treatment are structurally and functionally normal.

The primary goal of GSI treatment in chronic airway diseases would be to restore functional multiciliated cells to optimal numbers to improve mucociliary clearance, a vital host defense mechanism. We show that GSI treatment can increase multiciliated cell numbers after only a few days of treatment. LY450139 was able to drive multiciliated cell formation in differentiating as well as mature, homeostatic airway epithelial cultures, suggesting that GSI treatment can restore multiciliated cell numbers in both regenerating and intact portions of the airway epithelium. Importantly, GSIs increased multiciliated cells numbers in a dose-dependent manner, which suggests it may be possible to control the number of extra multiciliated cells that are generated. This is critical, as mucociliary clearance requires an optimal balance of multiciliated and secretory cells, and too many multiciliated cells could be detrimental to host defense. We show that LY450139 treatment during airway stem cell proliferation had no observable effect on subsequent differentiation, which suggests that it does not perturb the stem cell pool.

GSIs induce multiciliated cell formation by impinging on cellular differentiation programs, so it is likely to be a disease agnostic treatment for a wide range of chronic inflammatory diseases. In COPD and asthma, where aberrant Notch activation drives mucous cell hyperplasia (20, 21), GSI treatment would directly target this pathomechanism by blocking NOTCH receptor cleavage. We show that GSI treatment can also relieve IL-13-driven mucous cell hyperplasia. This likely occurs through the transdifferentiation of mucus secretory cells to multiciliated cells. Secretory club to multiciliated cell transdifferentiation has also been documented in mice lacking JAGGED ligand activity (24).

We previously showed that restoring multiciliated cells in CF primary cultures by GSI treatment led to improved barrier capacity as measured by transepithelial electrical resistance (TEER) (24). Notch is not known to directly regulate airway epithelial cell-cell junctions, but multiciliated cells express multiple unique apical junctional components (unpublished). This raises the possibility that optimal airway epithelial barrier function may also depend on having sufficient multiciliated cells. We also previously showed that GSI-treated CF cultures have improved epithelial scratch wound regeneration (24). The mechanism for this is unclear, but our combined data suggest that GSI treatment may broadly improve the structure and function of remodeled epithelia.

Despite the mitigation of remodeling phenotypes, chronic lung diseases will certainly still require other treatments such as antibiotics, anti-inflammatories, and/or CFTR modulators (e.g., E/T/I) in the case of CF. Thus, we propose GSIs as an adjuvant and not a stand-alone therapy. The majority of pwCF receive E/T/I as part of their standard of care treatment regimen (37). We show that in CF epithelial cultures treated with a combination of LY450139 and E/T/I, LY450139 did not interfere with the efficacy of CFTR-mediated Cl^-^ transport correction induced by E/T/I. We speculate that E/T/I may even lead to greater improvement of CF lung and sinus function in a structurally and functionally normalized epithelium resulting from adjunctive treatment with a GSI.

Due to their widespread and vital functions, therapeutic targeting of developmental signaling pathways such as Notch naturally carry the risk of on-target toxicity. Gastrointestinal toxicity of GSIs observed in clinical trials is thought to be related to Notch-dependent stem cell maintenance and differentiation in the gastrointestinal epithelium (39). Preclinical studies in Alzheimer’s mouse models demonstrated efficacy when LY450139 was administered at 30 mg/kg daily for 5 mo or at 100 mg/kg daily for 12 days, but not at 1 mg/kg daily for 8 days (40, 41). Human Phase I trials based on these and other nonclinical data were conducted at daily doses of 50 mg for 2 wk or 40 mg for 6 wk and at 100 mg and 140 mg daily for 14 wk, which showed that lower doses were well-tolerated, while higher doses were not well tolerated chiefly due to gastrointestinal adverse events (27, 28, 42). Development as an Alzheimer’s treatment was abandoned due to both lack of efficacy and unacceptable toxicities (26–28). We show that 1 mg/kg LY450139 administered daily for 3 days was sufficient to increase multiciliated cell numbers in mouse airways without any apparent toxicity. Chronic administration (once daily for five consecutive days per week for 3 wk) of this dose was also efficacious and well tolerated. Based on these data, we predict that efficacy at tolerated exposures may be achieved in humans with lower amounts of LY450139 dosed for a shorter duration. Furthermore, our data showing that apical treatment with LY450139 was just as efficacious as basal treatment in increasing multiciliated cells suggests that topical administration of LY450139 could be developed to potentially avoid systemic effects.

GSIs represent only one therapeutic option to block Notch signaling. Anti-NOTCH function-blocking antibodies are currently in clinical trials for solid tumors (26). A combination of anti-JAGGED1 and JAGGED2 antibodies has been shown to promote multiciliated cell formation in airway epithelial cells (43). Finally, inhibitors have been developed to target downstream signaling events, including IMR-1, which prevents the recruitment of Mastermind-like 1 to chromatin to block Notch target gene expression (44).

Finally, it is important to note that the molecular mechanism through which GSI treatment increases multiciliated cell numbers remains unclear. Mechanisms downstream of Notch ligand presentation are poorly understood in any system, although there is evidence that ligand-receptor complexes are internalized by the signal-sending cell after receptor cleavage, then are degraded (45). Internalization of this ligand-receptor complex may lead to activation of early ciliogenesis regulators; however, the increase in multiciliated cells when cleavage is blocked by GSIs argues against this. It is also possible that multiciliated cell fate acquisition is the default pathway in the airway epithelium unless Notch signaling is activated. This highlights a need for continued rigorous basic biology investigation of airway epithelial cell fate decisions to better understand this critical, potentially therapeutically important regulatory mechanism.

## DATA AVAILABILITY

Data will be made available upon reasonable request.

## SUPPLEMENTAL DATA

10.6084/m9.figshare.22304557 Supplemental Tables S1 and S2 and Supplemental Figs. S1–S4: https://doi.org/10.6084/m9.figshare.22304557.

## GRANTS

This work was mentored and financially supported by Stanford’s SPARK Translational Research Program. This project/publication was supported by the Stanford Maternal and Child Health Research Institute through Stanford’s SPARK Translational Research Program (to J.D.A., C.E.M., and E.K.V.), by an AP Giannini Postdoctoral Foundation Fellowship (to E.K.V.), a Cystic Fibrosis Foundation Research Grant (SELLER16L0, to Z.M.S.), and by the Ross Mossier CF Research Laboratory gift fund (to C.E.M.).

## DISCLOSURES

E.K.V. discloses the following related relationships/activities/interests: *1*) funding from Stanford Maternal and Child Health Research Institute through Stanford’s SPARK Translational Research Program and an AP Gianni Foundation Postdoctoral Fellowship and *2*) US Patent PCT/US2021046742. Z.M.S. discloses the following related relationships/activities/interests: *1*) funding from a Cystic Fibrosis Foundation Research Grant and *2*) consulting fees from Vertex Pharmaceuticals and AbbVie Inc. J.D.A. discloses the following related relationships/activities/interests: *1*) funding from Stanford Maternal and Child Health Research Institute through Stanford’s SPARK Translational Research Program and *2*) US Patent PCT/US2021046742. C.E.M. discloses the following related relationships/activities/interests: *1*) funding from Stanford Maternal and Child Health Research Institute through Stanford’s SPARK Translational Research Program and Ross Mosier CF Research Laboratories Gift Fund, *2*) consulting fees from Vertex Pharmaceuticals, *3*) US Patent PCT/US2021046742, and *4*) founder shares in Alentar BioSciences Inc. K.K., L.S.R.-H., J.M.S., N.S.J., and R.A.C. have no related relationships/activities/interests to disclose. None of the other authors has any conflicts of interest, financial or otherwise, to disclose.

## AUTHOR CONTRIBUTIONS

E.K.V., J.D.A., and C.E.M. conceived and designed research; E.K.V., K.K., L.S.R.-H., J.M.S., Z.M.S., N.S.J., R.A.C., J.D.A., and C.E.M. performed experiments; E.K.V., K.K., Z.M.S., N.S.J., R.A.C., J.D.A., and C.E.M. analyzed data; E.K.V., J.D.A., and C.E.M. interpreted results of experiments; E.K.V. prepared figures; E.K.V. drafted manuscript; E.K.V., Z.M.S., R.A.C., J.D.A., and C.E.M. edited and revised manuscript; E.K.V., K.K., L.S.R.-H., J.M.S., Z.M.S., N.S.J., R.A.C., J.D.A., and C.E.M. approved final version of manuscript.
